# Nanoparticle conjugation enhances the immunomodulatory effects of intranasally delivered CpG in house dust mite-allergic mice

**DOI:** 10.1038/srep14274

**Published:** 2015-09-21

**Authors:** Marie Ballester, Laura Jeanbart, Alexandre de Titta, Chiara Nembrini, Benjamin J. Marsland, Jeffrey A. Hubbell, Melody A. Swartz

**Affiliations:** 1 Institute of Bioengineering; 2 Swiss Institute for Experimental Cancer Research (ISREC); 3 Institute for Chemical Sciences and Engineering, Ecole Polytechnique Fédérale de Lausanne (EPFL), Switzerland; 4 Faculty of Biology and Medicine, Université de Lausanne (UNIL), Switzerland; 5 Institute for Molecular Engineering, University of Chicago, USA; 6 Materials Science Division, Argonne National Laboratory, USA

## Abstract

An emerging strategy in preventing and treating airway allergy consists of modulating the immune response induced against allergens in the lungs. CpG oligodeoxynucleotides have been investigated in airway allergy studies, but even if promising, efficacy requires further substantiation. We investigated the effect of pulmonary delivery of nanoparticle (NP)-conjugated CpG on lung immunity and found that NP-CpG led to enhanced recruitment of activated dendritic cells and to Th1 immunity compared to free CpG. We then evaluated if pulmonary delivery of NP-CpG could prevent and treat house dust mite-induced allergy by modulating immunity directly in lungs. When CpG was administered as immunomodulatory therapy prior to allergen sensitization, we found that NP-CpG significantly reduced eosinophilia, IgE levels, mucus production and Th2 cytokines, while free CpG had only a moderate effect on these parameters. In a therapeutic setting where CpG was administered after allergen sensitization, we found that although both free CpG and NP-CpG reduced eosinophilia and IgE levels to the same extent, NP conjugation of CpG significantly enhanced reduction of Th2 cytokines in lungs of allergic mice. Taken together, these data highlight benefits of NP conjugation and the relevance of NP-CpG as allergen-free therapy to modulate lung immunity and treat airway allergy.

Respiratory allergy is among the most common class of diseases in industrialized countries, with approximately 10% of the population affected today^1^. It can be characterized in two steps: (1) the sensitization phase, where allergen-specific CD4^+^ T cells are primed and produce the T helper 2 (Th2)-cytokines IL-4, IL-5 and IL-13, in turn inducing B cells to class switch and produce anti-allergen IgE^1^,^2^, and (2) the immediate or acute phase, whereby upon re-exposure to the allergen, allergens cross-link IgE on the surface of mast cells and basophils, leading to their degranulation and production of inflammatory factors^2^. Current treatments focus mainly on reducing symptoms (e.g., with corticosteroids and anti-histamines)^3^,^4^, although immunotherapeutic strategies that aim to reduce allergen hypersensitivity are emerging^1^. One immunotherapeutic strategy aims to desensitize allergic subjects to the allergen by repetitive administration of small allergen doses; however, this procedure is long, not always effective, involves extensive monitoring, and can have substantial systemic side-effects^1^,^5^. Another approach is allergen-free immunomodulation therapy, which aims to treat and reverse allergic symptoms and has the potential to be applied to a broad spectrum of allergies since it is not specific for one allergen^4^. These strategies utilize adjuvants such as Toll-like receptor (TLR) agonists with the premise that they may re-direct a pro-allergenic Th2-biased CD4^+^ T cell response toward a Th1-biased and thus less allergenic cytokine profile, an approach for which efficacy must be further substantiated^6^,^7^,^8^.

TLR agonists have differential effects on the lung environment and have been tested in treating airway allergy^1^. For example, moderate doses of the TLR3 ligand poly(I:C) and the TLR4 ligand LPS were able to induce allergen-specific IgE, while resiquimod, a TLR7/8 ligand, was not, making it a potential candidate for treatment of allergies^9^. In addition, CpG oligodeoxynucleotides have been investigated in allergen-desensitizing studies and have shown promise as allergen-free immunomodulating agents^4^,^10^. CpG engages TLR9 on B cells and dendritic cells (DCs), which are the main antigen-presenting cells in lungs^11^, and can potently induce Th1 immunity^12^ as well as downregulate established Th2 responses^4^,^10^,^11^,^13^. Additionally, TLR9 is widely expressed in the sinonasal mucosa^14^. Pre-clinical models of airway allergy and asthma have shown that CpG was able to reduce Th2 immunity, airway inflammation, eosinophilia, and IgE levels in both prophylactic and therapeutic settings^15^,^16^,^17^,^18^,^19^,^20^. While Th2 immunity is characteristic of allergic reactions and Th1 cytokines inhibit the synthesis of Th2 cytokines (the premise of immunomodulatory adjuvant therapy), it has been shown that immunotherapies can lead to the resolution of allergic reactions independently of Th1 markers^13^,^19^,^21^. The administration route of CpG is also important: pulmonary delivery, which targets the mucosa directly, was found to drive stronger effects of CpG on airway allergy symptoms compared to intradermal delivery^22^,^23^.

We have previously developed and described ultra-small (~30 nm) synthetic nanoparticles (NPs) able to target DCs in lymph nodes (LNs), both in skin-draining LNs after intradermal injection^24^,^25^,^26^,^27^ as well as in lung-draining LNs after pulmonary delivery^28^,^29^, and drive more potent DC maturation and antigen-specific T cell immunity when conjugated with CpG compared to free CpG^25^. Here, we hypothesized that NP-conjugated CpG-B (referred to hereinafter as NP-CpG), delivered via the pulmonary route, could enhance the activation of DCs in the lungs, induce strong and local Th1 immunity, and more effectively resolve airway allergy than free CpG. Other studies have demonstrated that combining CpG with delivery vehicles (including PLGA microparticles^30^ and virus-like particles (VLPs)^31^) or other adjuvants (e.g., mycobacterial extracts^18^) can enhance the targeting and efficacy of CpG in the context of airway allergy in animal models. However, further clinical evidence is needed to confirm effects on symptoms and our NPs may prove beneficial thanks to their size, delivery route and targeting properties.

To test this hypothesis, we began with a simple pulmonary vaccination model to demonstrate that NP-CpG with NP-antigen could induce an immune response with a strong Th1 bias. Then, we tested the efficacy of NP-CpG in both prophylactic and therapeutic models of airway allergy using the common aeroallergen house dust mite (HDM), which is a major source of allergens as well as bacterial and fungal products that can lead to airway allergy (allergic rhinitis and asthma) via TLR-4 triggering on airway structural cells^32^. HDM allergy is characterized by Th2-type inflammation, pulmonary eosinophilia, and airway remodeling^33^. In the prophylactic model of airway allergy, NP-CpG or free CpG was delivered the lungs of naïve mice before sensitizing and challenging them with HDM extract. We found that NP-CpG was significantly more effective than free CpG at reducing eosinophilia, IgE levels, mucus overproduction and Th2-related cytokine production. In a therapeutic setting where mice were first sensitized and then treated with NP-CpG, we found that although both free and NP-bound CpG could reduce eosinophilia and IgE levels, NP-CpG was significantly better at lowering Th2 cytokines. Together, these data highlight the potential of conjugating CpG to nanoparticles and their use in preventing and treating airway allergy in a clinical setting.

## Materials and Methods

### Mice

Female C57BL/6 mice (aged 8–12 weeks) were purchased from Harlan Laboratories (France) and kept under pathogen-free conditions at the animal facility of the EPFL. All experiments were performed in accordance with Swiss law and approved by the Cantonal Veterinary Office of the Canton de Vaud, Switzerland.

### Reagents

Chemicals were purchased from Sigma-Aldrich (Buchs, Switzerland). CpG-B 1826 ODN (5′-TCCATGACGTTCCTGACGTT-3′) and 5′SPO_3_-CpG-B 1826 were purchased from Microsynth (Balgach, Switzerland). Low endotoxin grade ovalbumin (OVA) (<0.01 EU/μg protein) (Hyglos, Regensburg, Germany) was used for immunizations and OVA grade VI (Sigma-Aldrich) was used for restimulations. Ground house dust mites (referred to simply as HDM) was obtained from Greer (Lenoir, NC, USA).

### Nanoparticle synthesis and conjugation

Pluronic-stabilized poly(propylene sulfide) NPs were synthesized, functionalized and surface-conjugated as previously described^26^,^27^. For antigen conjugation, OVA and NPs were incubated overnight at room temperature in the presence of guanidine hydrochloride (Sigma-Aldrich)^29^. For adjuvant conjugation, 5′ SPO_3_-modified CpG-B was incubated overnight in endotoxin free water (B Braun, Sempach, Switzerland) at room temperature^25^. NP-OVA and NP-CpG were purified by size-exclusion chromatography using CL6B matrix (Sigma-Aldrich), eluted and stored in PBS at room temperature. Protein concentration on NPs was determined by Pierce BCA protein assay (Thermo Scientific, Rockford, IL, USA) and CpG concentration on NPs was determined by GelRed (Brunschwig, Basel, Switzerland) assay. NP size before and after conjugation was determined by dynamic light scattering (Zetasizer, Nano ZS, Malvern, UK) to be of 30 ± 4 nm. All NPs formulations displayed endotoxin levels below 0.1 EU per administered dose, as detected with HEK-Blue hTLR4 cells from Invivogen (San Diego, CA, USA).

### Short-term Immunizations

After anesthesia with 5% isoflurane gas, mice were immunized by intranasal instillation via nostrils (also referred to as pulmonary delivery) with 5 μg OVA and 2 μg of free or NP-conjugated CpG in a volume of 50 μl. For DC activation and analysis of cytokines in the bronchoalveolar lavage (BAL), mice were sacrificed 24 h later. For T cell readouts, mice were immunized again on day 14 and sacrificed on day 19 post-immunization.

### House dust mite-induced airway inflammation and immunomodulation

For the pre-treatment study, mice were pre-treated on days 1, 3, and 5 with 2 μg CpG (free or NP-conjugated) by pulmonary delivery, and then sensitized on days 8, 10, and 12 with 15 μg HDM. They were then challenged with 15 μg HDM on days 22, 24, and 26, and sacrificed on day 30. For the therapeutic regime, mice were first sensitized with 15 μg HDM on days 1, 3, and 5, then treated with 2 μg NP-CpG or free CpG on days 15, 17, and 19, and finally challenged with 15 μg HDM on days 22, 24, and 26; mice were sacrificed on d 30. All solutions were prepared in PBS, adjusted to a 50 μl volume and were instilled intranasally for delivery to the lungs under isoflurane anesthesia.

### Collection and analysis of bronchoalveolar lavage

The bronchoalveolar lavage (BAL) was collected by flushing the airways three times with 1 ml PBS. Differential BAL cell counts were performed on cytospins stained with a Diff Quick staining set (Siemens Healthcare Diagnostics, Zurich, Switzerland). Total numbers and percentages of eosinophils, macrophages, lymphocytes and neutrophils were determined microscopically using standard morphological and cytochemical criteria. Two hundred cells were counted on each slide and frequencies of macrophages, neutrophils, eosinophils and lymphocytes were calculated with respect to the total number of BAL cells on each slide. The number of cells per cell type was back calculated by multiplying the % of cells in each population by the total number of cells collected in the BAL of each mouse. We report both parameters (frequencies and cell numbers) to account for the distribution of cells (% of total), for the recruitment to the airways (total number), and for their relative impact on each other.

### Cell preparation and *ex vivo* restimulation

LNs were digested 30 min in IMDM medium (Invitrogen, Zug, Switzerland) supplemented with 1 mg/ml collagenase D (Roche, Rotkreuz, Switzerland). Single cell suspensions were obtained by gently disrupting LNs through a 70 μm strainer, followed by washing with HBSS. Lungs were perfused with 10 ml PBS and digested 45 min and processed as described above. Afterwards, a 30% Percoll (VWR, Nyon, Switzerland) gradient was applied to the cells to isolate lung leukocytes.

For T cell restimulation followed by intracellular staining and flow cytometry, cells were incubated for 6 h at 37 °C in the presence of 100 μg/ml OVA protein with the addition of 5 μg/ml Brefeldin A (Sigma-Aldrich) for the last 3 h of culture. All cells were cultured in IMDM supplemented with 10% fetal bovine serum (FBS) and 1% penicillin/streptomycin (P/S) (all from Invitrogen). For T cell restimulation followed by ELISA, cells were incubated for 3d at 37 °C in the presence of 100 μg/ml OVA protein in IMDM medium supplemented with 10% FBS and 1% P/S (all from Invitrogen).

### Preparation of lung homogenates

Lung lobes were collected and transferred to lysing matrix D tubes (MP Biomedicals, Santa Ana, CA, USA) with 300 μl lysis buffer solution composed of 15 ml T-PER tissue protein extraction reagent (Thermo Scientific) supplemented with a complete EDTA-free proteinase inhibitor tablet (Roche Diagnostics, Mannheim, Germany). Lungs were homogenized and centrifuged, and supernatant was collected and stored at −20 °C for later analysis by ELISA.

### Flow cytometry

Prior to antibody staining for flow cytometry, cells were labeled with live/dead fixable cell viability reagent (Invitrogen). For intracellular cytokine staining, cells were fixed in 2% paraformaldehyde in PBS, washed with 0.5% saponin (Sigma-Aldrich) in staining buffer (PBS with 2% FBS) and incubated with the antibodies diluted in the same saponin solution. After washing, cells were resuspended in staining buffer for analysis. Samples were acquired on CyAn ADP Analyzer (Beckman Coulter, Brea, CA, USA) and data was analyzed with FlowJo software (Tree Star Inc., Ashland, OR, USA). Antibodies against mouse CD11c, CD80, CD86, CD4, and IFN-γ were from Biolegend (San Diego, CA, USA), antibodies against mouse CD3, TNF-α, IL-2 and MHC-II were from eBioscience (San Diego, CA, USA).

### ELISA

Ready-SET-go! ELISA kits for IFN-γ, IL-4, IL-5, IL-6, IL-10, IL-12p70, IL-13, IL-17a and TGF-β1 (all from eBioscience) were used according to manufacturer’s instructions. IgE ELISA reagents (kind gift of Nicola Harris, EPFL), including IgE standard grown from TIB-141 (ATCC, Manassas, VA, USA), IgE unlabeled coating antibody grown from 6HD5, IgE biotin (Biolegend) and streptavidin-AP (Southern Biotech, Birmingham, AL, USA) were used as previously described^34^.

### Histology, mucus and inflammation scores

Lungs were flushed with 1 ml neutral 4% buffered formalin solution. The left lobes of lungs were then embedded in paraffin and stored overnight at 4 °C, sectioned, and stained with periodic-acid Schiff (PAS) reagents (Sigma) according to standardized protocols. Sections were imaged on a slide-scanning microscope (VS120-L100, Olympus, Tokyo, Japan) and analyzed with Fiji software (NIH, Bethesda, MD USA). Mucus score was quantified as the average number of mucus-producing goblet cells per unit length of bronchi circumference. The inflammation score for each mouse was quantified as the inflamed surface area minus the area of the blood vessel lumen, normalized to the average inflamed surface area minus the area of blood vessel lumen in naïve mice.

### Statistical analysis

Statistically significant differences between experimental groups were determined by one-way ANOVA with Bonferroni multiple comparison post-test using Prism software (GraphPad, San Diego, CA, USA). Statistics were performed between allergic groups, treated and untreated where *P < 0.05, **P < 0.01 and excluded naive mice, unless expressly specified where ^#^P < 0.05, ^##^P < 0.01 compared to naïve mice.

## Results

### Pulmonary NP-CpG activates lung-resident DCs and leads to Th1 immunity in lungs

**Figure 1:**
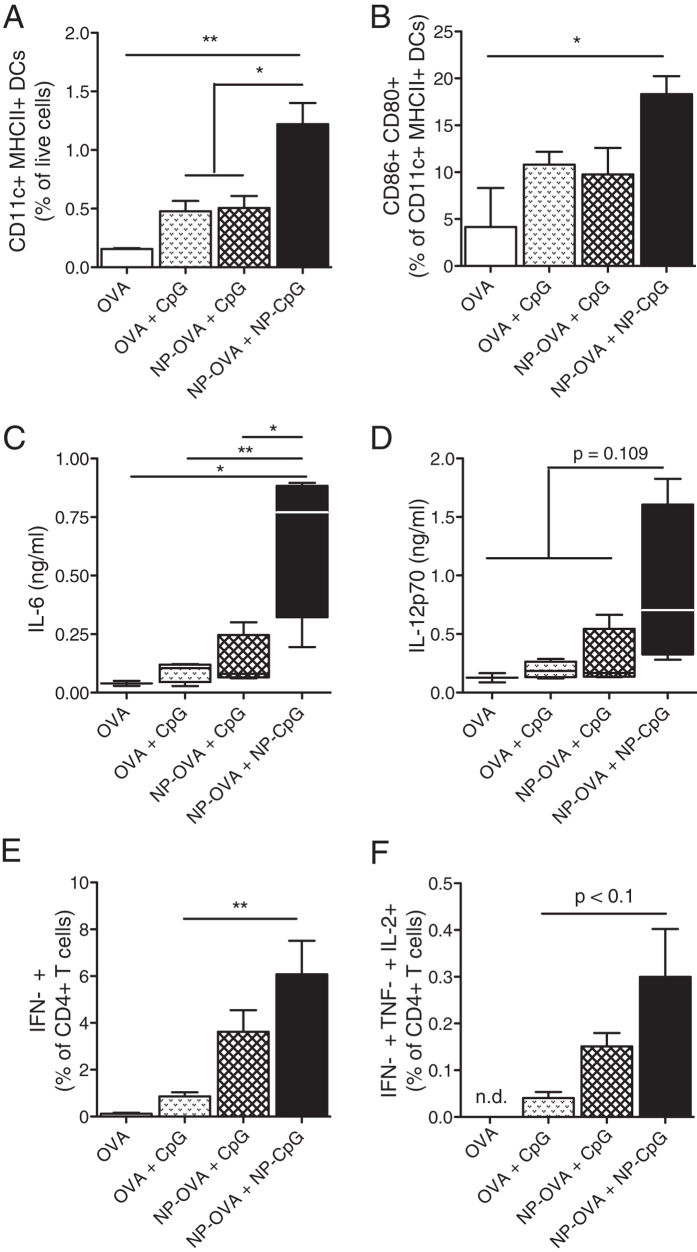
Pulmonary NP-CpG activates lung-resident DCs and leads to Th1 immunity in lungs. Mice received 5 μg OVA and 2 μg CpG (free or NP-conjugated) via the pulmonary route and (**A–D**) sacrificed 24 h later or (**E,F**) immunized again on day 14 and sacrificed on day 19. (**A**) Frequencies of all dendritic cells (DCs, CD11c^+^ MHCII^+^) in the lungs, as percentages of live cells. (**B**) Co-expression of the activation markers CD86 and CD80 (as percentages of CD11c^+^ MHCII^+^ DCs). (**C**) IL-6 and (**D**) IL-12p70 concentrations in the bronchoalveolar lavage (BAL) as determined by ELISA. (**E**) IFN-γ^+^ T cells and (**F**) polyfunctional (IFN-γ^+^ TNF-α^+^ IL-2^+^) T cells in lungs after restimulation with OVA (as percentages of CD4^+^ T cells). Data show mean ± SEM from two independent experiments, 7 mice per group (2 mice in OVA group). *P < 0.05, **P < 0.01.

To assess whether NP-CpG delivered via the pulmonary route can affect the activation of lung-resident DCs, mice were immunized with a model vaccine consisting of 5 μg OVA and 2 μg CpG, free or NP-conjugated, in a solution of 50 μl delivered via the pulmonary route. NP-CpG co-delivered with NP-OVA led to significantly higher frequencies of CD11c^+^ MHCII^+^ DCs in the lungs 24 h post-instillation compared to free CpG (Fig. 1A), and these DCs had a more activated phenotype as seen by co-expression of CD80 and CD86 (Fig. 1B) compared to DCs in the OVA-treated group; DCs in the free CpG immunized mice were not more activated than DCs in the OVA group. NP-conjugated CpG also led to significantly more IL-6 secretion in the BAL as compared to mice immunized with free CpG (Fig. 1C); differences in IL-12p70 were not statistically significant (Fig. 1D). For T cell read-outs, mice received a boost 14 days later and were sacrificed on day 19. Upon *ex vivo* restimulation with OVA and subsequent intracellular staining for flow cytometric analysis, CD4^+^ T cells from lungs of mice immunized with NP-OVA + NP-CpG expressed significantly more IFN-γ than cells from mice immunized with OVA + CpG (Fig. 1E). Furthermore, we observed a trend towards more CD4^+^ T cells co-expressing IFN-γ, TNF-α and IL-2 when immunized with NP-OVA + NP-CpG than with other vaccines (Fig. 1F), showing not only enhanced Th1 immunity but also a polyfunctional phenotype of T cells in the lungs when CpG was conjugated to NPs. Thus, this data shows that NP-conjugation of CpG and delivery to lungs activates lung DCs and skews local immunity towards a Th1 phenotype more efficiently than free CpG.

### Pre-treatment with NP-CpG reduces eosinophilia and IgE levels in lungs of allergic mice

**Figure 2:**
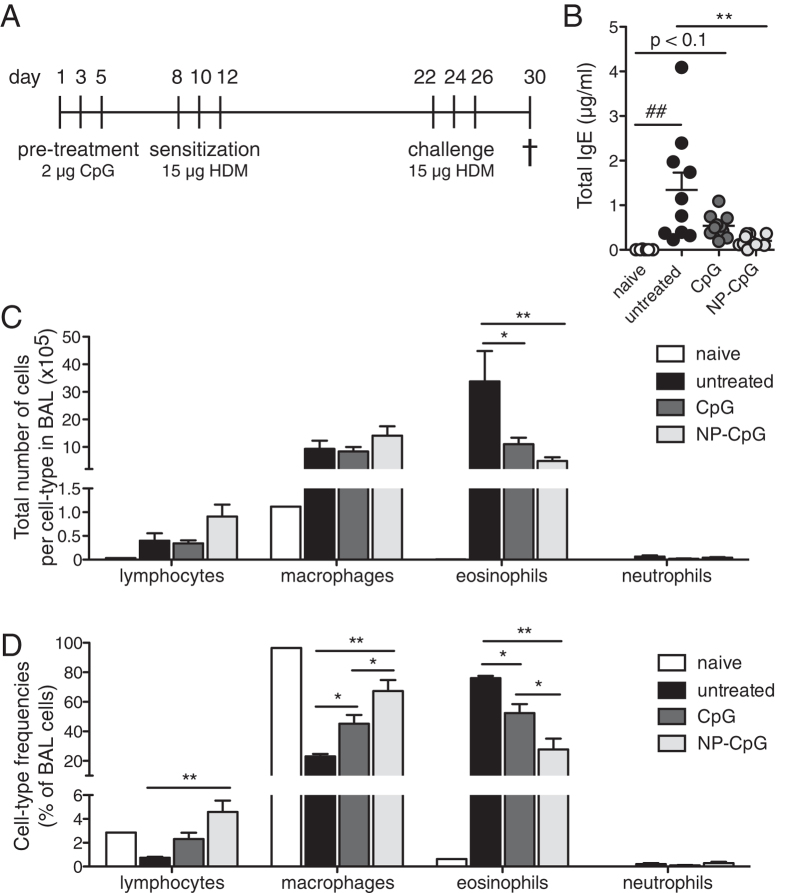
Pre-treatment with NP-CpG reduces eosinophilia and IgE levels in lungs of allergic mice. Mice were pre-treated with 2 μg CpG (free or NP-conjugated) on days 1, 3, and 5. They were then sensitized with 15 μg HDM on days 8, 10, and 12. Finally, they were challenged with 15 μg HDM on days 22, 24, and 26, and sacrificed on day 30. All solutions were delivered via the pulmonary route. (**A**) Experimental timeline. (**B**) Total IgE levels in serum at day 30 as determined by ELISA. (**C**) Numbers and (**D**) frequencies of lymphocytes, macrophages, eosinophils and neutrophils in bronchoalveolar lavage (BAL) as defined by Diff Quick staining of cytospins. Data show mean ± SEM from two independent experiments, 10 mice per group, *P < 0.05, **P < 0.01; ^##^P < 0.01 compared to naïve mice.

To investigate the efficacy of NP-conjugated CpG in preventing allergies, we used a model of HDM allergy and delivered CpG via the pulmonary route as allergen-free immunomodulatory prophylaxy (shown in Fig. 2A). We found that amongst the treatment groups, NP-CpG significantly reduced IgE levels compared to that in untreated mice, and free CpG did not reduce IgE levels compared to untreated mice; as such, NP-CpG was more effective at reducing IgE levels than free CpG (Fig. 2B); in fact, NP-CpG levels were not different from those in naïve mice (Fig. 2B). Furthermore, NP-CpG significantly lowered the total eosinophil counts in the BAL compared to untreated mice (Fig. 2C), and also decreased the frequency of eosinophils and increased the frequency of macrophages compared to free CpG (Fig. 2D). Free CpG also decreased eosinophilia while increasing frequencies of macrophages compared to untreated mice, but not to the same extent that NP-CpG did (Fig. 2D). Taken together, these results indicate that prophylactic treatment with NP-CpG, but not free CpG, could significantly impact IgE and eosinophilia resulting from allergen challenge.

### Pre-treatment with NP-CpG recruits DCs and decreases Th2 cytokines in lungs of allergic mice

**Figure 3:**
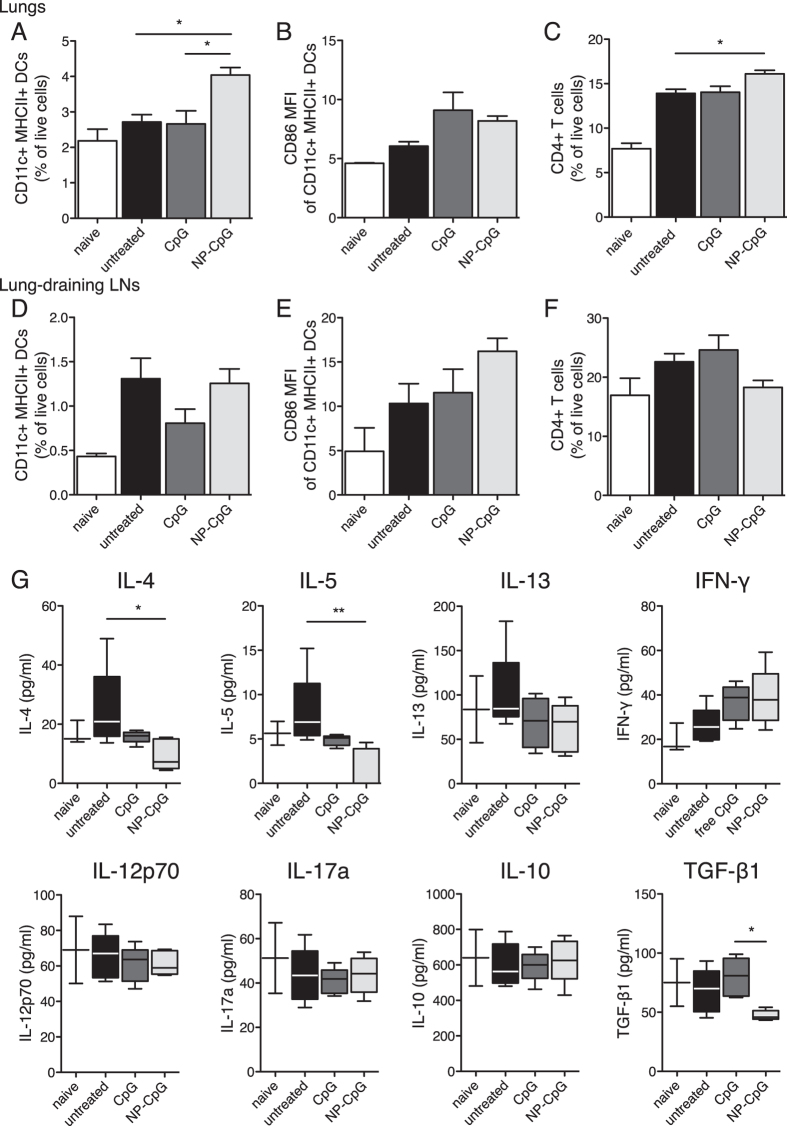
Pre-treatment with NP-CpG recruits DCs and decreases Th2 cytokines in lungs of allergic mice. Mice were pre-treated with CpG and then sensitized and challenged with HDM as in Fig. 2A. Analysis of dendritic cells (DCs) and CD4^+^ T cells in the lungs (**A**–**C**) and lung-draining lymph nodes (LNs) (D-F): (**A,D**) frequencies of all CD11c^+^ MHCII^+^ DCs as percentage of live cells, (**B**,**E**) relative expression levels (mean fluorescence intensity, MFI) of CD86 expression by CD11c^+^ MHCII^+^ DCs, and (**C,F**) frequencies of CD4^+^ T cells (as percentage of live cells). (**G**) Concentrations of indicated cytokines in lung homogenates as determined by ELISA. Data represent mean ± SEM from two independent experiments, 10 mice per group. *P < 0.05, **P < 0.01.

In addition to decreasing eosinophilia, mice pre-treated with NP-CpG before sensitization and challenge with HDM (Fig. 2A) displayed significantly more CD11c^+^ MHCII^+^ DCs in the lungs than untreated mice or mice pre-treated with free CpG (Fig. 3A). DCs in lungs of CpG-pre-treated mice, free or NP-conjugated, had a slightly more activated phenotype as determined by CD86 expression (Fig. 3B). Finally, NP-CpG pre-treated mice had significantly higher frequencies of CD4^+^ T cells in the lungs than untreated mice (Fig. 3C). The effects of CpG on DCs and T cells were locally limited to cells in lungs, as free and NP-conjugated CpG had no statistically significant impact on DCs and CD4^+^ T cells in lung-draining LNs (Fig. 3D–F). Of note, NP-CpG had no statistically significant, long-term effect on DCs in lungs and lung-draining LNs following pulmonary delivery without allergen exposure (Supplementary Figure). We hypothesized that CpG may affect the Th2 phenotype classically associated with allergy, and we analyzed cytokines present in lung homogenates of allergic and pre-treated mice. Immunomodulation with NP-CpG led to significantly lower IL-4 and IL-5 secretions as compared to untreated mice (Fig. 3G). CpG, free and NP-bound, led to a moderate decrease in IL-13 and increase in IFN-γ, while having no effect on IL-12p70, IL-17a and IL-10 levels as compared to untreated allergic mice. NP-CpG also led to a significant decrease in TGF-β1 secretion as compared to other mice (Fig. 3G). Taken together, this data indicates that NP-CpG as pre-treatment leads to a long-lasting effect on DCs, that more CD4^+^ T cells get recruited to lungs and that these CD4^+^ T cells may lose their Th2 phenotype as seen by lower IL-4, IL-5 and IL-13 levels.

### Pre-treatment with NP-CpG reduces mucus production in lungs of allergic mice

**Figure 4:**
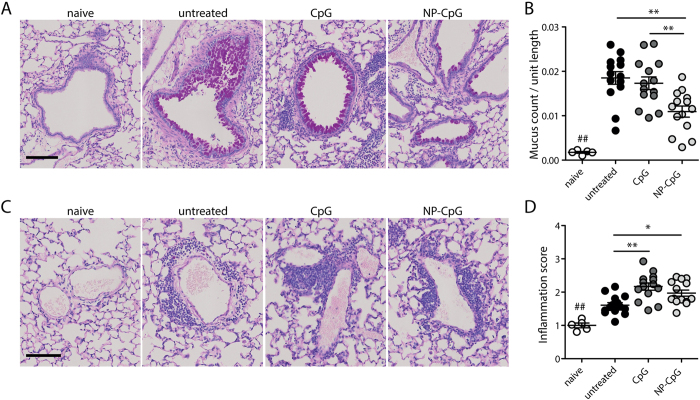
Pre-treatment with NP-CpG reduces mucus production in lungs of allergic mice. Mice were pre-treated with CpG and then sensitized and challenged with HDM as in Fig. 2A. (**A**) Representative periodic acid-Schiff (PAS)-stained lung sections showing mucus-producing goblet cells (dark magenta) within bronchi (scale bar, 100 μm). (**B**) Quantification of mucus-producing goblet cells per unit length. (**C**) Representative PAS-stained lung sections showing leukocyte accumulation (blue) around blood vessels (scale bar, 100 μm). (**D**) Inflammation score, defined as area of leukocyte infiltration and normalized to the average value in naïve mice. Data show mean ± SEM from three independent experiments, 14 mice per group (5 mice in naïve group). *P < 0.05, **P < 0.01; ^##^P < 0.01 compared to naïve mice.

Following the same experimental timeline as in Fig. 2A, we found that NP-CpG significantly prevented or lowered mucus overproduction in bronchi as compared to either untreated allergic mice or mice pre-treated with free CpG, as observed and quantified from lung sections stained with periodic acid-Schiff (PAS) to reveal mucus-producing goblet cells (Fig. 4A,B). Both free CpG and NP-CpG led to increased leukocyte infiltration around blood vessels (Fig. 4C) compared to untreated allergic mice, indicative of ongoing immune responses in the lungs (Fig. 4D). These data indicate that while both CpG and NP-CpG drove similar levels of leukocyte infiltration into the lung, only NP-CpG was able to reduce mucus overproduction in bronchi of allergic mice, a major symptom of airway inflammation.

### Therapeutic delivery of NP-CpG reduces IgE, eosinophilia, and Th2 immunity in lungs of allergic mice

**Figure 5:**
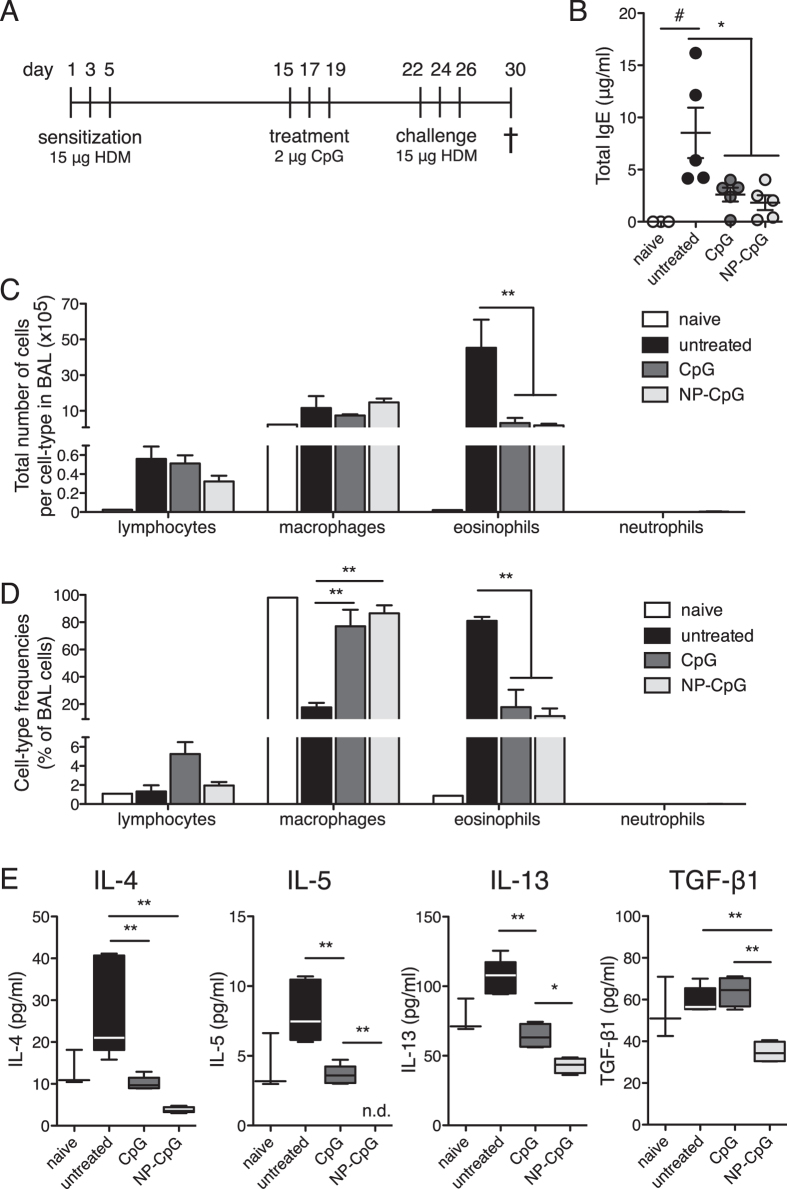
Therapeutic delivery of NP-CpG reduces IgE, eosinophilia, and Th2 immunity in lungs of allergic mice. Mice were first sensitized with 15 μg HDM on days 1, 3, and 5, then treated with 2 μg CpG (free or NP-conjugated) on days 15, 17, and 19, then challenged with 15 μg HDM on days 22, 24, and 26, and finally sacrificed on d 30. All solutions were delivered via the pulmonary route. (**A**) Experimental timeline. (**B**) Total IgE concentrations in serum at day 30 as determined by ELISA. (**C**) Numbers and (**D**) frequencies of macrophages, eosinophils, lymphocytes and neutrophils in the bronchoalveolar lavage (BAL) as defined by Diff Quick staining of cytospins. (**E**) Concentrations of indicated cytokines in lung homogenates as determined by ELISA. Data show mean ± SEM, 5 mice per group. *P < 0.05, **P < 0.01; ^#^P < 0.05 compared to naïve mice.

After demonstrating efficacy of pulmonarily delivered NP-CpG in preventing airway allergy, we sought to investigate its potential therapeutic efficacy in allergy immunomodulation. We considered a situation analogous to that in seasonal environmental allergy, where patients have already encountered allergens from a previous season, and could potentially be treated in the spring before pollen appearance, for example. In this scenario, mice were first sensitized to HDM to mimic pre-existing allergy, then treated with CpG or NP-CpG, and finally challenged with HDM (Fig. 5A). Interestingly, we found that when delivered therapeutically, both free CpG and NP-CpG significantly and substantially reduced serum IgE levels (Fig. 5B) and eosinophilia in the BAL (Fig. 5C,D) of allergic mice as compared to untreated mice. Furthermore, both free CpG and NP-CpG similarly increased frequencies of macrophages and lymphocytes in the BAL (Fig. 5D). Finally, both free CpG and NP-CpG significantly and substantially reduced lung IL-4, IL-5 and IL-13 levels in lung homogenates (Fig. 5E), but NP-CpG reduced IL-5 and IL-13 secretions to significantly lower levels than did free CpG, and only NP-CpG could reduce TGF-β1 levels in allergic mice (Fig. 5E). Taken together, these results show that although both CpG and NP-CpG delivered in a therapeutic setting to allergic mice could reduce allergy markers (IgE, eosinophilia), NP-CpG was significantly more potent than free CpG overall at reducing Th2 immunity.

## Discussion

In this study, we sought to improve efficacy of adjuvant therapy for airway allergy by using a delivery system designed to target lung-draining LNs and lung-resident cells. Our ultrasmall polymeric NPs were previously shown to efficiently drain to LNs and target immature DCs there^24^ and were more effective as vaccine formulations when delivered to the lung compared to unconjugated vaccine counterparts^35^. As such, they demonstrated superior Th1-mediated protection against pathogenic challenges, for example in models of tuberculosis^28^ and influenza^29^. Since the TLR9 agonist CpG had demonstrated promise in allergen-free immunotherapy against allergy in previous studies^4^,^7^,^10^, we therefore hypothesized that by improving its targeting to lung-resident DCs and lung LNs, conjugation to ultrasmall NPs could allow the same dose of CpG to yield greater efficacy in preventing and treating airway allergy.

In naïve mice, we found that pulmonary delivery of NP-conjugated CpG enhanced DC recruitment and activation, in turn driving effector CD4^+^ T cells and skewing the lung environment towards Th1 immunity. When given as allergen-free immunomodulatory prophylaxy in a model of HDM allergy, NP-CpG was significantly and substantially more potent than free CpG at reducing IgE levels, eosinophilia, mucus production, and Th2 cytokines. In a therapeutic setting, NP-CpG reduced Th2 cytokines significantly more than free CpG, and both treatments reduced IgE levels and eosinophilia. These results highlight the efficacy of NP-CpG administered via the mucosal (pulmonary) route in modulating the lung environment and also the potential therapeutic benefits of using NP-CpG in preventing and treating airway allergies.

Because DCs internalize allergens and play a key role in inducing and controlling Th2 immunity in allergic patients, it is desirable to modulate DC function to prevent or treat sensitized subjects with immunomodulators^36^,^37^. Furthermore, we observed previously that 24 h after delivery more DCs and fewer macrophages took up NPs in lungs when co-delivered with free CpG than without^29^, which led us to hypothesize that conjugating CpG to NPs would recruit and activate DCs in lungs. We first showed that conjugating CpG to NPs enhanced DC recruitment to lungs 24 h post-pulmonary delivery (Fig. 1). In our model of allergy pre-treatment, we found that frequencies of DCs in lungs remained more elevated 30 days post-treatment with NP-CpG than with free CpG (Fig. 3). However, in a long term study where mice were immunized with NP-CpG without allergen exposure (Supplementary Figure), NP-CpG did not impact DCs differently from free CpG or NPs only: DC frequencies and activation status were similar in immunized as in naïve lungs and lung-draining LNs. We have previously showed that NPs are present in mucosal tissues 12 and 72 h post intranasal instillation, that the amount of NPs peaks at 12 h, and goes down at 72 h^35^, which leads us to speculate that NPs are cleared within a few days of administration. Taken together, this suggests that the long-term effect of NP-CpG observed in the allergy pre-treatment model is due to a combination of NP-CpG and allergen and that this immunomodulation happens locally in lungs.

In a prophylactic model of lung immunomodulation prior to allergen sensitization and challenge, NP-CpG lowered serum IgE levels—down to naïve levels—and eosinophilia in lungs more profoundly than did free CpG (Fig. 2). We hypothesized that the enhanced efficacy of NP-CpG was due to TLR9 activation on DCs, induction of Th1-skewed immune responses and thus dampened Th2 responses^10^. IL-4 and IL-5 play central roles in the initiation and perpetuation of Th2 immunity and allergy^38^: B cells cannot class-switch to produce IgE without IL-4^1^,^6^, and eosinophils cannot home to and become activated in lungs without IL-5^2^,^6^. Consistent with this hypothesis, IL-4 and IL-5 were significantly reduced in lungs of mice pre-treated with NP-CpG compared to those with free CpG (Fig. 3), hence supporting the premise that NP-CpG is a more potent inhibitor of Th2 immunity than free CpG. Furthermore, NP-CpG significantly increased the recruitment of CD4^+^ T cells in the lungs, and these T cells lost their Th2 phenotype as compared to untreated mice. However, other mechanisms might come into play to reduce Th2 responses, such as the differential recruitment and activation of DCs when CpG is conjugated to NP, leading to a modified lung environment^39^. Other studies with CpG have also shown that IFN-γ and other Th1 markers were sometimes but not always elevated and necessary to reduce Th2 responses^18^,^19^,^21^. With NP-CpG, we aimed at skewing the lung environment away from exacerbated Th2 responses, but without inducing a negative, albeit counter-acting, immune response. Enhanced Th1 responses might be detrimental, however here we show that we do not induce exacerbated Th1 immunity, and we believe this is not the only mechanism with which NP-CpG dampens Th2 responses in our model. Although CpG is known as a Th1-immunity inducer, CpG could decrease Th2 immunity in airway allergy without inducing strong Th1 immunity or Th1 cytokines^15^,^19^,^21^; for example CpG could promote the expression of MHC II, CD80 and CD86 on DCs which can then activate T cells and skew them away from a Th2 phenotype independently from IFN-γ. In addition, sensitized epithelial cells and eosinophils are major sources of TGF-β1, which is involved in mucus production and airway remodeling in asthmatic patients^40^. Finally, IL-5 and IL-13 have been linked to airway TGF-β1^40^. Taken together, lower TGF-β1 levels in lungs with NP-CpG (Figs. 3 and 5) may be a consequence of lower eosinophilia and Th2-related cytokine production (IL-4, IL-5, IL-13) in NP-CpG treated mice.

When delivered prior to induction of allergy, NP-CpG-driven responses led to a decrease in mucus production by goblet cells (Fig. 4), a major symptom of airway allergy due to several factors secreted during inflammation^41^,^42^. Lymphocyte aggregation around blood vessels was seen with CpG and NP-CpG (Fig. 4), indicating ongoing immune responses that are consistent with the immunomodulatory properties of CpG^7^,^12^,^43^.

In the therapeutic setting (Fig. 5), where mice were sensitized to HDM prior to CpG therapy, NP-CpG reduced the Th2 cytokines IL-4, IL-5 and IL-13, and TGF-β1 significantly more than free CpG, which itself reduced levels as compared to untreated mice (Fig. 5). It has been suggested that eosinophils are dispensable for allergic remodeling in HDM allergic mice, which may suggest that Th2 immunity is a more important parameter to monitor and reduce^44^. As a strong immune-modulatory molecule, CpG may have been able to redirect CD4^+^ T cells away from a Th2 phenotype as seen by lower IL-4, IL-5 and IL-13 production. Hammad *et al.* showed that inflammatory DCs were necessary and sufficient for induction of Th2 immunity to inhaled HDM and that depletion of eosinophils had no effect on Th2 responses^45^. This suggests that NP-CpG, which better targets DCs than free CpG, had a direct and stronger effect on DCs and on subsequent Th2 cytokine production and that this effect may be separate from an effect on eosinophils.

NP-CpG and free CpG reduced IgE levels and eosinophilia to a similar extent in the therapeutic setting (Fig. 5). Because IgE production and allergen cross-linking lead to mast cell activation and subsequent recruitment of eosinophils, the lack of difference between free CpG and NP-CpG effects on eosinophils may stem from the lack of difference in IgE levels in treated allergic mice. Furthermore, lower secretions of IL-4, IL-5, and IL-13 induced by NP-CpG and CpG might affect *de novo* B cell class-switching and IgE production, possibly explaining the lack of difference seen between CpG and NP-CpG treatments on IgE levels (Fig. 5). Given the long-term effects of CpG on the immune system^46^, we also hypothesize that NPs may further prolong the effect of CpG in the lungs. The effect of NP conjugation may not yet translate to differential therapeutic benefits in the lungs (Fig. 5) thus explaining similar reductions in IgE and eosinophil levels with free CpG and NP-CpG in the therapeutic setting where mice were sacrificed 2 weeks post-CpG treatment as opposed to 4 weeks post-CpG delivery in the prophylactic setting.

The delivery route and CpG dose are key parameters to optimize therapeutic benefits and we observed striking effects on allergy markers after delivering a small dose (2μg) of CpG when coupled to NPs via the pulmonary route. CpG doses of 10–100 μg led to equivalent or lower reductions of allergy symptoms in different mouse studies^16^,^18^,^19^,^23^, indicating a clear advantage for the NP system to effectively deliver small doses of CpG-hence potentially reducing toxicity – and maximize immunity modulation. Mucosal (pulmonary) vaccination is superior to parenteral (intradermal, intraperitoneal) delivery to induce mucosal and local immunity to CpG in lungs^22^,^23^,^28^,^47^ and this study is one of the few exploring the pulmonary route of administration for allergy immunotherapy^6^,^8^,^15^,^16^,^18^,^31^,^48^,^49^.

The fully synthetic NPs carry CpG-B on their surface and release this cargo intracellularly upon reduction of the disulfide bond linking CpG to NPs^25^,^27^. These features make the PEG-PPS NPs attractive for potential translation in terms of manufacturing and safety compared to other nanocarriers. For example, virus-like particles (VLPs), which have been investigated with CpG-A in allergy pre-clinical and clinical studies and failed to meet endpoints^31^,^50^,^51^, are not synthetic but are rather composed of viral proteins^52^. VLPs carry their cargo in their core^6^,^50^,^53^, so while both nanocarriers have an optimal size (20–50 nm) for uptake by antigen-presenting cells in the respiratory tract^54^,^55^,^56^, CpG on the surface of NPs may be more readily available to TLR9 in endosomes^25^,^53^ compared to the encapsulated CpG within VLPs. Furthermore, CpG-B has been demonstrated to induce stronger B cell proliferation, DC activation and IL-12p70 secretion compared to CpG-A^25^,^43^, making CpG-B an interesting alternative to CpG-A for therapeutic purposes. As such, although VLPs failed to meet primary endpoints in a recent phase IIb clinical trial for asthma, NPs offer a promising alternative for the delivery and use of CpG in airway allergies.

In conclusion, we show that pulmonary administration of NP-conjugated CpG was significantly more effective than free CpG at reducing key allergy symptoms in both prophylactic and therapeutic settings of HDM-induced airway allergy and we speculate that NP-CpG may be a valid therapeutic candidate for airway allergies. This study shows the benefits of using NPs as a delivery platform to enhance the activity of immunomodulators administered via the mucosal route to lungs and suggests the further development of our NP platform to prevent and treat airway allergies in the clinic.

## Additional Information

**How to cite this article**: Ballester, M. *et al.* Nanoparticle conjugation enhances the immunomodulatory effects of intranasally delivered CpG in house dust mite-allergic mice. *Sci. Rep.* **5**, 14274; doi: 10.1038/srep14274 (2015).

## Supplementary Material

Supplementary Figure
