# Supplementary material for: Nanoparticle conjugation enhances the immunomodulatory effects of intranasally delivered CpG in house dust mite-allergic mice
Source: Sci Rep. 2015 Sep 21;5:14274. doi: 10.1038/srep14274 (PMC4585708; doi:10.1038/srep14274)
Supplement: Supplementary Figure [file srep14274-s1.pdf]

Nanoparticle conjugation enhances the immunomodulatory effects of intranasally delivered CpG in house dust mite-allergic mice

Authors:

Marie Ballester*[1], Laura Jeanbart*[1,2], Alexandre de Titta[1], Chiara Nembrini[1], Benjamin J. Marsland[4], Jeffrey A. Hubbell[1,3,5,6], Melody A. Swartz[1,2,3,5]

Author affiliations:

[1]Institute of Bioengineering, [2]Swiss Institute for Experimental Cancer Research (ISREC), and [3]Institute for Chemical Sciences and Engineering; Ecole Polytechnique Fédérale de Lausanne (EPFL), Switzerland

[4]Faculty of Biology and Medicine, Université de Lausanne (UNIL), Switzerland

[5]Institute for Molecular Engineering, University of Chicago, USA

[6]Materials Science Division, Argonne National Laboratory, USA

*These authors contributed equally to this work.

Corresponding author:

Melody A. Swartz, Institute of Bioengineering, School of Life Sciences and Engineering, EPFL, SV IBI LLCB, AI 1106, Lausanne, 1015, Switzerland. Phone: +41 21 693 9686; Fax: +41 21 693 9685; E-mail: melody.swartz@epfl.ch

Supplementary Information

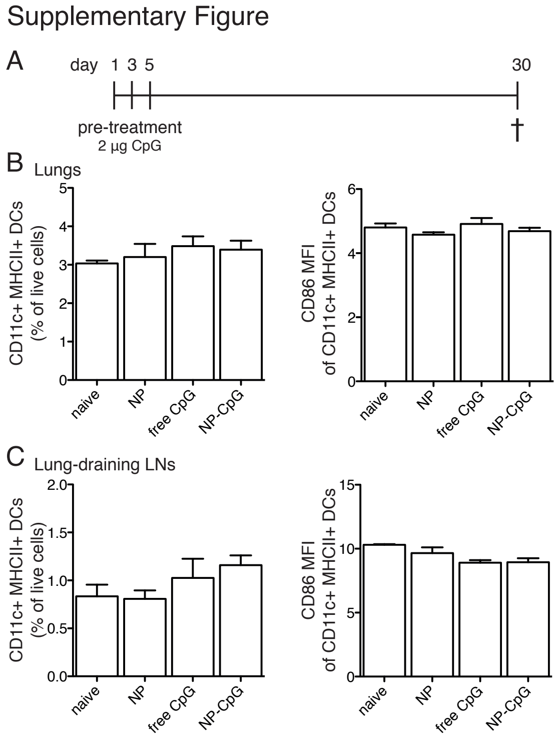

Supplementary Figure. NP-CpG does not have a long-term effect on DCs in lungs and lung-draining lymph nodes following pulmonary delivery

Mice received 2 µg CpG (free or NP-conjugated) via the pulmonary route on days 1, 3, and 5. Mice were sacrificed on day 30. The NP group received an equivalent dose of NPs only as in the NP-CpG group. (A) Experimental timeline. Analysis of dendritic cells (DCs) in the lungs (B) and lung-draining LNs (C): (*left*) frequencies of $CD11c^+$ $MHCII^+$ DCs as percentage of live cells, (*right*) relative expression levels (mean fluorescence intensity, MFI) of CD86 expression by $CD11c^+$ $MHCII^+$ DCs. Data represent mean ± SEM, 4-5 mice per group.
